# Tracking westerly wind directions over Europe since the middle Holocene

**DOI:** 10.1038/s41467-022-34952-9

**Published:** 2022-12-21

**Authors:** Hsun-Ming Hu, Valerie Trouet, Christoph Spötl, Hsien-Chen Tsai, Wei-Yi Chien, Wen-Hui Sung, Véronique Michel, Jin-Yi Yu, Patricia Valensi, Xiuyang Jiang, Fucai Duan, Yongjin Wang, Horng-Sheng Mii, Yu-Min Chou, Mahjoor Ahmad Lone, Chung-Che Wu, Elisabetta Starnini, Marta Zunino, Takaaki K. Watanabe, Tsuyoshi Watanabe, Huang-Hsiung Hsu, G.W.K. Moore, Giovanni Zanchetta, Carlos Pérez-Mejías, Shih-Yu Lee, Chuan-Chou Shen

**Affiliations:** 1 High-Precision MassSpectrometry and Environment Change Laboratory (HISPEC), Department of Geosciences, National Taiwan University, Taipei, 10617 Taiwan ROC; 2 Research Center for Future Earth, National Taiwan University, Taipei, 10617 Taiwan ROC; 3 Laboratory of Tree-Ring Research, University of Arizona, Tucson, AZ 85721 USA; 4 Institute of Geology, University of Innsbruck, 6020 Innsbruck, Austria; 5 Université Côte d’Azur, CNRS, CEPAM, 06300 Nice, France; 6 Université Côte d’Azur, CNRS, OCA, IRD, Géoazur, 06560 Valbonne, France; 7 Department of Earth System Science, University of California, Irvine, CA 92697-3100 USA; 8 HNHP, UMR 7194, Sorbonne Universités, MNHN, CNRS, UPMC, UPVD, Paris, France; 9 Laboratoire Nice-Côte d’Azur, Fondation IPH, 06300 Nice, France; 10 Department of Geography Science, Fujian Normal University, 350117 Fuzhou, Fujian China; 11 College of Geography Science, Nanjing Normal University, 210046 Nanjing, China; 12 Department of Earth Sciences, National Taiwan Normal University, Taipei, 11677 Taiwan ROC; 13 Department of Ocean Science & Engineering, Southern University of Science and Technology, 518055 Guangdong, China; 14 Department of Geography and Environmental Sciences, Northumbria University, Newcastle, NE1 8ST UK; 15 Department of Civilizations and Forms of Knowledge, University of Pisa, 56126 Pisa, Italy; 16 Archaeological Superintendency of Liguria, 16126 Genova, Italy; 17 Toirano Cave, 17055 Toirano, Italy; 18 Department of Natural History Sciences, Faculty of Science, Hokkaido University, Sapporo, 060-0810 Japan; 19 KIKAI Institute for Coral Reef Sciences, Kikai Town, 891-6151 Japan; 20 Research Center for Environmental Changes, Academia Sinica, Taipei, 11529 Taiwan ROC; 21 Department of Physics, University of Toronto, Toronto, ON M5S 1A7 Canada; 22 Department of Chemical and Physical Sciences, University of Toronto Mississauga, Mississauga, ON L5L 1C6 Canada; 23 Department of Earth Sciences, University of Pisa, Pisa, 56100 Italy; 24 CIRSEC, Centre for Climatic Change Impact, University of Pisa, Pisa, Italy; 25 INGV, Pisa, 56125 Italy; 26 IGAG-CNR, Monterotondo, Rome, Italy; 27 Institute of Global Environmental Change, Xi’an Jiaotong University, 710049 Xi’an, China

**Keywords:** Climate sciences, Hydrology

## Abstract

The variability of the northern westerlies has been considered as one of the key elements for modern and past climate evolution. Their multiscale behavior and underlying control mechanisms, however, are incompletely understood, owing to the complex dynamics of Atlantic sea-level pressures. Here, we present a multi-annually resolved record of the westerly drift over the past 6,500 years from northern Italy. In combination with more than 20 other westerly-sensitive records, our results depict the non-stationary westerly-affected regions over mainland Europe on multi-decadal to multi-centennial time scales, showing that the direction of the westerlies has changed with respect to the migrations of the North Atlantic centers of action since the middle Holocene. Our findings suggest the crucial role of the migrations of the North Atlantic dipole in modulating the westerly-affected domain over Europe, possibly modulated by Atlantic Ocean variability.

## Abstract (web summary)

Combined with other westerly-sensitive records, a new stalagmite hydroclimate record from northern Italy reveals changing westerly wind directions over the past 6500 years that correspond to a migration of the North Atlantic centres of action.

## Introduction

The Mediterranean Basin has been a cradle of civilizations since the middle Holocene (8.2–4.2 thousand years before 1950 C.E., kyr BP). Today some 400 million people live in this region. As a “climate hot spot,” the Mediterranean features large hydroclimate variability in response to global climate change^1^ and has been experiencing exceptionally low rainfall over the past two decades^2^, resulting in significant impacts on ecosystems, human society, and the economy^3^. The drought has been attributed to an enhanced sea-level pressure (SLP) contrast between the Azores High and the Icelandic Low (the SLP dipole, hereafter; Supplementary Fig. 1), known as the positive phase of the North Atlantic Oscillation (NAO)^4^, which led to a northerly migration of the westerlies, transporting moisture away from the Mediterranean region (Supplementary Fig. 2a). The location of the SLP dipole is not stable through time. Its spatial displacement determines the angle of the westerly tracks and the phase of the East Atlantic (EA) pattern^5–11^, defined by a pressure anomaly centered over the eastern North Atlantic (52°30’N, 27°30’W; Supplementary Fig. 2b)^12^. The variabilities of the SLP dipole and varying westerly tracks can therefore result in unstable NAO-correlated rainfall regimes (red and blue areas in Supplementary Movie 1) in Europe on decadal to multidecadal timescales, termed “non-stationary NAO” behavior^13^.

Climate proxy records are essential to develop an in-depth understanding of the westerlies on a range of timescales. For example, terrestrial and marine sediments in Iberia^14^, and Moroccan tree-ring data combined with Scottish speleothem records^15^ suggest a prolonged positive NAO phase during the Medieval Climate Anomaly (1050–850 yr BP). However, Northern Hemisphere proxy assemblages and model simulations argued that proxy-based paleo-NAO reconstructions are sometimes contentious^16–18^, largely because of the complex interactions among Atlantic SLP. Only few proxy records reliably reflect Atlantic SLP patterns, rendering tracking the westerlies’ position across mainland Europe challenging^19^. Since NAO-correlated rainfall regimes (Supplementary Movie 1) reflect the position of the westerlies^5–11^, past positions of the westerlies can be constrained by comparing a series of westerly-sensitive records in the North Atlantic region.

Here, we present a multi-annually resolved precipitation record from such a sensitive region (northern Italy). In conjunction with other regional westerly-sensitive archives, these data depict large-scale hydroclimate changes and the variability of the SLP dipole and the westerlies over the past 6500 years.

## Results and discussion

Bàsura cave (44°08′N, 8°12′E; Supplementary Figs. 1 and 3 and Supplementary Text 1) is located in Toirano, Liguria, northern Italy, an area characterized by a typical Mediterranean climate. More than 70% of the annual precipitation, 1276 (± 310) mm (1σ, 1833–2008 C.E.), falls during the rainy seasons from September to February (Genoa meteorological station; 44°24’N, 8°05’E; 56 m above sea level; Supplementary Fig. 3a). The 1-km-long cave has a mean annual cave air temperature of 15.6 °C (2013–2014 C.E.) and 97–100% relative humidity beyond 100 m from the entrance. Two stalagmites, BA14-1 and BA18-4, were collected at sites with 98–100% relative humidity (Supplementary Figs. 3b and 4). Their U-Th-based age model (Supplementary Fig. 5) shows that they cover the time interval from 6437 ± 12 to 5 ± 18 yr BP. About 1000 subsamples were extracted along the growth axis of the stalagmites for δ^18^O analysis (Supplementary Figs. 4 and 6) to establish a composite δ^18^O record (∆^18^O; Supplementary Fig. 6; Methods). Hendy tests^20^ (Supplementary Figs. 4 and 7) of 13 growth layers suggest that calcite precipitated at near isotopic equilibrium (Supplementary Text 2). In combination with the coeval Sr/Ca data (Supplementary Text 3 and Supplementary Fig. 8; Methods), the ∆^18^O is interpreted as a westerly-sensitive record, with negative/positive values corresponding to strong/weak westerlies over northern Italy and high/low rainfall amount (Supplementary Text 2).

The decadal to multi-centennial westerly fluctuations since 6.5 kyr BP documented by the Bàsura ∆^18^O record are consistent with regional reconstructions, largely reflecting hydroclimate changes (Supplementary Text 2). This ∆^18^O series is in good agreement with a lacustrine record^21^ and a stalagmite-based wet/dry index from Italy^22,23^ as well as hydroclimate reconstructions from Spain^24^, Portugal^25^, and Algeria^26^ (Supplementary Fig. 9 and Supplementary Table 2). For example, the correlation coefficients of Bàsura ∆^18^O with lacustrine flood records from northern Italy (Supplementary Fig. 9a)^21^ and cave records from central Italy (Supplementary Fig. 9b, c)^22,23^ are –0.42 (*n* = 31, *p* < 0.05) and 0.67 (*n* = 31, *p* < 0.05), respectively (Supplementary Table 2). The drought between 5 and 4 kyr BP was concurrent with dry periods recorded by speleothems from Turkey^27^, Lebanon^28^, Israel^29^, and Morocco^30,31^ (Supplementary Fig. 10a–d). The following wet period during 4–3 kyr BP is in good agreement with records from circum-Mediterranean countries (Supplementary Figs. 9d–f, 10a, b, and 11c, d)^24–28,32,33^. These events likely had a profound impact on ancient Mediterranean human societies (Supplementary Fig. 12). For example, the 5.2 kyr BP dry period has been suggested to have resulted in the demise of the Uruk period^29^, while the dry period at 4.8–4.1 kyr BP (4.2-kyr event) possibly induced the collapse of the Old Kingdom^34^ and the Akkadian Empire^35^.

Hydroclimate changes in northern Italy are strongly affected by North Atlantic SLP variability (Supplementary Fig. 2a, b) and westerly dynamics. Instrumental precipitation data from Genoa (Genoa PP) are positively/negatively correlated with the 850-mb zonal wind around Gibraltar/Iceland during the rainy season (Supplementary Fig. 2c), suggesting that changes in the position of the westerlies affect Genoa PP. Genoa PP show a strong negative correlation with SLP variations centered over northwestern Europe (Supplementary Fig. 2d), resembling the EA pattern^12^, implying that spatial changes of the SLP dipole dominate regional rainfall patterns. The SLP in northwestern Europe (averaged over [45–55°N, 20°W–0]) is also significantly anti-correlated with the 850-mb zonal wind around Toirano (averaged over [42–47°N, 5–15°E]), with a correlation coefficient of −0.6 ± 0.04 (*n* = 97; *p* < 0.01; 1920–2016 C.E.) during winter (December–February, DJF) based on NCAR/NCEP Reanalysis v3. This anticorrelation emphasizes that the westerlies dominate the hydroclimate in Toirano. The leading empirical orthogonal function (EOF) 1 of European-Atlantic (60°W–40°E, 20–80°N) precipitation represents an EA teleconnection (Supplementary Fig. 13a) that is positively correlated with Genoa PP (*r* = 0.43 ± 0.04, *n* = 173, *p* < 0.01). Combined with the NAO pattern observed in EOF2 (Supplementary Fig. 13b), our results show that the variability of the SLP dipole influences Toirano precipitation (Supplementary Fig. 2a, b). The precipitation patterns documented by the Toirano records hence reflect movements of the westerlies under the two climate modes, consistent with previous studies^36–38^.

**Fig. 1:**
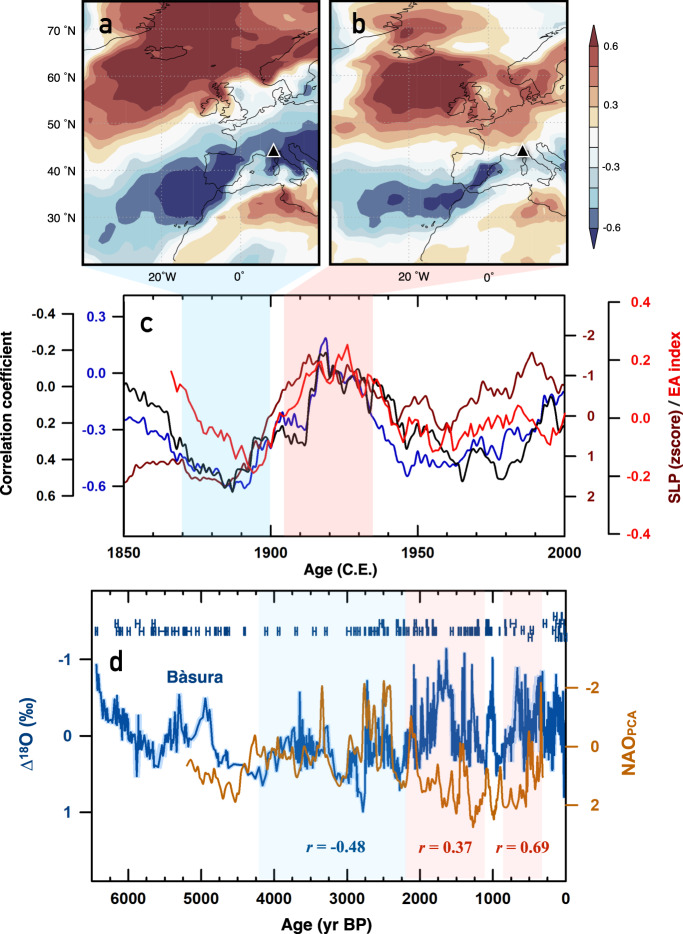
Decadal to millennial non-stationary North Atlantic Oscillation (NAO) behaviors. **a** Correlation of winter (December-February) precipitation and NAO index^39^ during 1870–1900 C.E. Note that the reanalysis of periods before 1920 C.E. is not as reliable as those after 1920 C.E. due to the lack of observation data in NCAR/NCEP Reanalysis v3. **b** as **a**, but for 1905–1935 C.E. Color-coded shadows in **a** and **b** show the correlation coefficient. Black triangle edged white marks the location of Bàsura cave. **c** Black: 30-year window running correlation between December-March (DJFM) Toirano precipitation (averaged in [45–48°N, 5–10°E]) and W Greenland temperature (averaged in [63–68°N, 60–50°W]); blue: 30-year window running correlation between DJFM Toirano precipitation (averaged in [45–48°N, 5–10°E]) and NAO index^39^; brown: 10-year moving average of DJFM sea-level pressure (SLP) at [52.5°N, 27.5°W]); red: 10-year moving average of reconstructed DJF East Atlantic (EA) index^41^. The sign of this EA index is inverted in order to match the EA definition used in this study. The y-axis for the blue series is reversed compared to the other two. **d** Bàsura ∆^18^O record (blue) and a lacustrine record from western Greenland^40^ (brown). The light-blue shadow in the background of Bàsura ∆^18^O shows the ranges of 2-sigma age uncertainties. The correlation coefficient in **d** is inverted due to the inverse relationship between Bàsura ∆^18^O and precipitation (Supplementary Text 2). Horizontal bars are U-Th dates with 2-sigma errors. Cyan/pink shaded areas denote the climate conditions resembling negative/positive EA. Data source in **a–c**: NCAR/NCEP Reanalysis v3 (https://www.esrl.noaa.gov/). Maps of **a** and **b** are generated using KNMI climate explorer (https://climexp.knmi.nl/).

On multi-decadal to multi-centennial time scales, Bàsura stalagmite-inferred paleo-precipitation is positively correlated (*r* = 0.69, *n* = 292, 99%, Methods) with a NAO reconstruction^16^ of the past millennium (1049–1969 C.E.; Supplementary Fig. 14). In contrast to the negative correlation of modern Genoa PP with the NAO index^39^ in winter (DJF; Supplementary Fig. 2a), the comparison suggests that Toirano was in a positive NAO-correlated region over the past millennium, where the westerlies were strong and precipitation was high during the positive NAO phase. When compared with lacustrine sediment records from western Greenland^40^ (Fig. 1d) that reflect NAO-driven local temperature changes and westerly dynamics, Bàsura ∆^18^O inferred precipitation patterns also show a positive correlation in the interval 2.2–1.2 kyr BP (*r* = 0.37, *n* = 64, 95%) and 0.8–0.3 kyr BP (*r* = 0.69, *n* = 41, 95%), but a negative correlation (*r* = –0.48, *n* = 84, 99%) between 4.2 and 2.2 kyr BP (Fig. 1d). In other words, the relationship of Toirano precipitation with the reconstructed NAO index^16^ remained positive after 2.2 kyr BP, but was negative in the interval 4.2–2.2 kyr BP, revealing that the NAO-correlated regions over Europe have changed since the middle Holocene.

### Non-stationary North Atlantic Oscillation

Over the past 150 years, correlations of Toirano precipitation with western Greenland temperature (Fig. 1c; black line) and with the NAO index^39^ (Fig. 1c; blue line) have been unstable, reflecting the non-stationary NAO behavior (Supplementary Movie 1). Correlations between NAO index and Toirano precipitation were, for example, more significant for 1870–1900 and 1950–1980 C.E. than for 1905–1935 C.E. (Fig. 1c), suggesting that Toirano was located in negative NAO-correlated regions in the late 19^th^ and late 20^t^^h^ centuries (Fig. 1a), but close to the boundary of (or even temporally in positive) NAO-correlated regions in the early 20^th^ century (Fig. 1b). Such changes in NAO-correlated regions have been suggested to be modulated by the migrations of the SLP dipole (i.e., EA phases)^5–11^. Northward or counterclockwise rotated displacements of the SLP dipole could lead the westerly tracks towards a southwest-northeast tilt and shift the positive NAO-correlated regions towards higher latitudes^5,9^ (e.g., Fig. 1a).

**Fig. 2:**
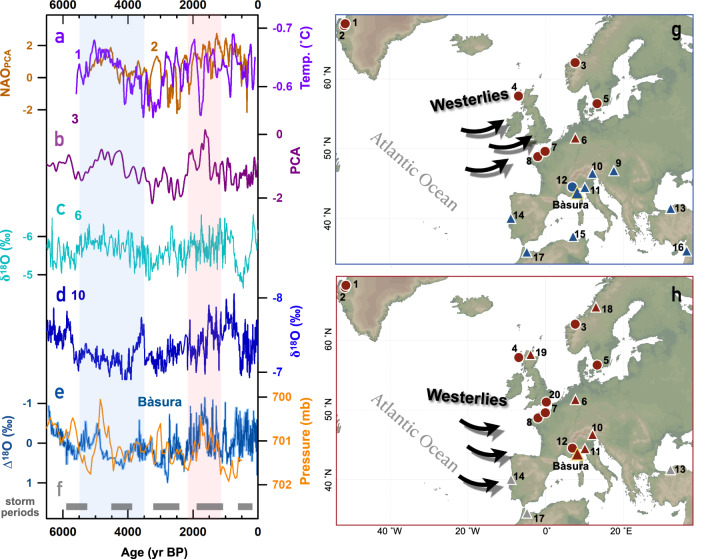
Migration of the westerlies. **a** The reconstructed North Atlantic Oscillation (NAO_PCA_) index^40^ (brown) and Greenland lake temperature (temp.) reconstructions^43^ (purple). Low temperature indicates positive NAO phase^43^. **b** Principal component analysis (PCA) based glacier activity reconstructions from alpine lake sediments^46^ in Norway. High value of PCA represents small glaciers and a warm climate. **c** Stalagmite δ^18^O records from Bunker cave, Germany^45^. **d** Stalagmite δ^18^O from Spannagel cave^44^, Austria. Negative δ^18^O values in **c** and **d** represent mild and wet conditions. **b** and **d** are detrended to remove the effect of neo-glaciation in the middle to late Holocene. **e** Blue: Bàsura ∆^18^O record. The light-blue shadow in the background of Bàsura ∆^18^O shows 2-sigma age uncertainties. Orange: Simulated 700-mb height pressure at [10°W–10°E, 45–50°N] from TraCE-21ka, UCAR (http://www.cesm.ucar.edu/). **f** Composite record documenting major Holocene storminess periods in northern Europe^49^. Numbers at records in **a**–**f** are sites in **g** and **h**. **g** Schematic figure showing the direction of the westerlies and westerly-affected regions during 5.4–3.5 kyr BP as inferred in this study. **h** As **g**, but for 2.2–1.2 kyr BP. Cyan/pink shaded areas denote the conditions resembling negative/positive EA. The triangles/circles denote the locations of the cave/sediment records with numbers showing their corresponding reference below. Their colors indicate wet/warm (red), cold/dry (blue), and conditions difficult to categorize (gray). Maps in **g** and **h** were generated using Ocean Data View. **1**: Lake SS1220^40^. **2**: Braya So/Lake E^43^. **3**: Nerfloen Lake^46^. **4**: Scotland costal dunes^47^. **5**: Undarsmosse bog^48^. **6**: Bunker cave^45^. **7**. Seine Estuary^49^. **8**: Mont-Saint Michel Bay^49^. **9**: Trio cave^32^. **10**: Spannagel cave^44^. **11**: Corchia cave^21,23^. **12**: Lake Savine^21^. **13**: Sofular cave^27^. **14**: Buraca Gloriosa^25^. **15**: Gueldaman cave^26^. **16**: Jeita cave^28^. **17**: Charra cave and Piste cave^30,31^. **18**: Korallgrottan cave^50^. **19**: Roaring cave^52^. **20**: Scotney Marsh^51^ (Supplementary Table 1).

Variable correlations between Toirano precipitation and hydroclimate in the North Atlantic (Fig. 1c; black and blue lines) match the trend of the SLP time series over southwestern England (Fig. 1c; brown line) and the reconstructed EA index (Fig. 1c; red line)^41^. This confirms that EA phase changes could affect the locations of the SLP dipole and hence the NAO-correlation regions over Europe, in accordance with previous studies^9^. For longer timescales, isotope-enabled general circulation models indicate that the multi-decadal correlation patterns of NAO-precipitation δ^18^O in Europe can be affected by the EA phases^42^. The Bàsura ∆^18^O also shows similarity with the 700-mb height pressure variation in the eastern Atlantic on centennial to millennial scales obtained from a transient simulation (TraCE-21ka) with all forcings using the Community Climate System Model version 3 (Fig. 2e). Accordingly, correlation changes between the NAO index and Bàsura ∆^18^O (Fig. 1d) over the past thousands of years can be attributed to the variability in the position of the SLP dipole, which modulates the NAO-correlation regions.

Our conclusions are supported by the comparisons with westerly-sensitive records across Europe. For example, during the positive NAO phase 5.4–3.5 kyr BP^40,43^ (Fig. 2a), proxy records reveal a cool/dry climate in the regions below ~45°N^21–31,44^ (Fig. 2d, e, g and Supplementary Figs. 9 and 10a–d) and a wet/warm climate in northern Europe^45–49^ (Fig. 2b, c, g). However, during another positive NAO phase 2.2–1.2 kyr BP^40,43^ (Fig. 2a), southern^21,24^ (Fig. 2e), central^44^ (Fig. 2d and Supplementary Fig. 9b, d), and northern Europe^45–52^ (Fig. 2b, c) were characterized by multi-centennial wet and warm conditions, while this period featured ambiguous hydroclimate fluctuations in the eastern Mediterranean^27–29^ (Supplementary Fig. 10a–c) and northern Africa^31,53^ (Supplementary Fig. 10d, e). In addition, intense storminess in northern Europe^49^ coincides with droughts in southern Europe during 5.8–5.5, 4.5–3.95, and 3.3–2.4 kyr BP (Fig. 2f) but with wetness in southern Europe during 1.9–1.05 and 0.6–0.25 kyr BP. These observations suggest that the NAO-correlated regions over Europe and the North Atlantic westerly tracks changed. A running correlation analysis (Supplementary Fig. 15) between Bàsura ∆^18^O and proxy records from Norway^46^, Germany^45^ and Austria^44^ reveals that Norway and Germany were in a different precipitation domain than Bàsura cave before versus after ~3 kyr BP. Austria, on the other hand, remained in the same precipitation domain as Bàsura cave over the entire period. The shifts of the SLP dipole might be time-transgressive and an exact time boundary is difficult to define, but in general, the SLP dipole migrated southward after 2.2 kyr BP. This is supported by European pollen-based wet/dry reconstructions^54^ and is consistent with a southward migration of the oceanic Azores front between 6.5 kyr BP and the late Holocene^55^.

The observation of the changing NAO-correlated regions demonstrates a centennial to millennial mixing effect of NAO and EA modes on European hydroclimate changes. The NAO mode in principle dominated the “dipole” precipitation pattern over mainland Europe (Fig. 1a, b) over the past 6500 years. The EA mode also played an important role in modulating the centennial to millennial positions of the NAO dipole. This phenomenon confirms the combined effect of NAO and EA on positions of the NAO dipole over the instrumental period^8,9^. For example, a concurrent positive NAO phase and negative EA phase during the period 5.4–3.5 kyr BP (Fig. 2g) forced the Azores High northeastward. Coinciding positive NAO and EA phases from 2.2–1.2 kyr BP, on the other hand, resulted in a southwestward shift of the Azores High.

### Migration of the westerlies since the middle Holocene

SLP dipole movements could alter the route of the westerlies over the East Atlantic^5–11^. A northward displacement of the Azores High, such as prior to 2.2 kyr BP, favors a SW-NE direction of the westerlies over Europe. In this case, the pivot point of the SLP dipole (the NAO-zero correlation line) can also rotate into a SW-NE direction, restricting the positive NAO-correlated regions to northwestern Europe (Fig. 2g). After 2.2 kyr BP, the SLP dipole migrated southwards and the westerlies tilted less over Europe. The positive NAO-correlated regions shifted southward and northern Italy was thus positively correlated with the reconstructed NAO index (Fig. 2h).

A high degree of similarity between our Bàsura ∆^18^O record and a stalagmite δ^18^O series from Kaite cave, Spain (Supplementary Fig. 9d)^24^ strengthens our argument of the relationship between westerly routes and the variability of the SLP dipoles since the middle Holocene. Variability in the Kaite cave δ^18^O record reflects the zonal migration of the Icelandic Low and the positive δ^18^O values are related to westward shifts of the Icelandic Low. Positive Bàsura ∆^18^O values, on the other hand, are associated with an eastward migration of the Azores High. The similar patterns of Bàsura ∆^18^O and Kaite δ^18^O (Supplementary Fig. 9d), therefore, suggest the co-occurrence of westward shifts of the Icelandic Low with eastward shifts of the Azores High. The induced counterclockwise rotation of the SLP dipole in turn can lead to a change in the NAO-correlated regions over Europe^8,9^. Combined with studies that suggest more frequent positive NAO phases from the middle to the late Holocene^40,56,57^, our results reveal the complex interaction between the NAO state and its dipole locations on centennial to millennial scales, with associated changes in the orientation of westerly routes across Europe.

### Possible effect of latitudinal oceanic thermal gradient on the sea-level pressure dipole

**Fig. 3:**
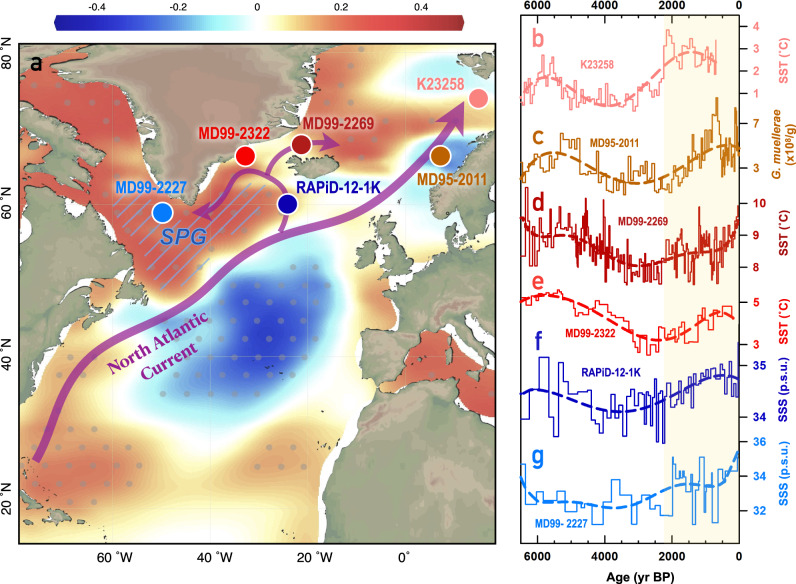
Atlantic sea-surface conditions in the last 6500 years. **a** The North Atlantic Current (purple arrows) and the locations of cited records (white-edged circles). Reb/blue areas show the inverse correlation coefficients between 700-mb height pressure variations in the eastern Atlantic (52°30’N, 27°30’W) and sea-surface temperatures (SST) from a 300-year preindustrial controlled Community Earth System Model 2 (CESM2; Methods)^66^. Dotted regions indicate that the correlations are significant at the 95% confidence level. Blue slashed region denotes the location of the Subpolar Gyre (SPG). Map was generated using Ocean Data View. **b**, **d**, and **e** SST records from K23258^60^ (N Norway), MD09-2269^61^ (N Iceland), and MD99-2322^62^ (E Greenland), respectively. **c** *G. muellerae* abundance as a proxy of Atlantic water inflow in Nordic Sea^65^. **f** and **g** Sea-surface salinity (SSS) records from RAPiD-12-1K^63^ (southern Iceland) and MD99-2227^64^ (Labrador Sea), respectively. Dashed lines in **b-g** denote their 6^th^ order trend lines. Yellow shaded rectangle marks the period with increasing North Atlantic Current influence at high latitudes.

The movements of the SLP dipole are likely modulated by the latitudinal gradient of sea-surface temperatures (SSTs) in the North Atlantic. On decadal to centennial scales, observational reanalysis and model simulations have shown that the multidecadal SLP response to Atlantic Multidecadal Variability (AMV) projects on migrations of the SLP dipole (i.e., changes in EA phases)^58,59^. Warm AMV phases that feature a warm North Atlantic Ocean correspond to a southward shift of the SLP dipole (positive EA). Over the past 2.2 kyr, SSTs have increased in northern Norway^60^ (K23258, Fig. 3b) and eastern Greenland^61,62^ (MD99-2269, Fig. 3d; MD99-2322, Fig. 3e), in line with elevated sea-surface salinity (SSS) levels around the Subpolar Gyre^63,64^ (Fig. 3a; RAPiD-12-1K, Fig. 3f; MD99-2227, Fig. 3g) and an increased proportion of Atlantic water-sensitive coccoliths in the Nordic Sea^65^ (MD95-2011, Fig. 3c). These observations reveal that, over the past 2.2 kyr, more warm, salty, low-latitude Atlantic water has been delivered towards high latitudes compared to before, possibly reducing the latitudinal SST gradient and hence leading to a southward migration of the SLP dipole. The linkage of AMV and EA is also in accord with results of the preindustrial controlled Community Earth System Model 2 (CESM2)^66^ (Methods), which shows a strong coherence between SLP anomalies over the East Atlantic and SST changes along the North Atlantic Current (Fig. 3a), suggesting that Atlantic Ocean dynamics play an important role in the migration of the SLP.

Our ∆^18^O series in combination with other westerly-sensitive records show multiple patterns of European westerly drift on decadal to millennial scales since the middle Holocene in response to the non-stationary behavior of the NAO that was possibly modulated by Atlantic sea-surface conditions. Our results underscore the impact of changing Atlantic Ocean circulations on the position of the westerlies over Europe that in the near future might be associated with variability of the meridional overturning circulation, increasing greenhouse gases, and/or varied aerosol concentrations^59^.

## Methods

### Stalagmite samples, U-Th dating, and age model

Two stalagmites, 190-mm-long BA14-1 and 90 mm-long BA18-4, were collected in two cave chambers of narrow side passages of Bàsura cave, 500 and 800 m from the entrance (Supplementary Fig. 3a), in January 2014 and June 2018, respectively. The stalagmites were cut into halves and polished (Supplementary Fig. 4). High resolution scanning electron microscope (SEM) analysis shows that BA14-1 formed as needle-shaped aragonite and BA18-4 as rhombic calcite crystals (Supplementary Fig. 4).

A total of 78 (BA14-1) and 18 (BA18-4) subsamples, 10–100 mg each, were drilled for U-Th chemistry^67^ and dating^67,68^ (Supplementary Data 1) at the High-Precision Mass Spectrometry and Environment Change Laboratory (HISPEC), Department of Geosciences, National Taiwan University. All U-Th isotopic measurements were conducted on a multi-collector inductively coupled plasma mass spectrometer, Thermo-Finnigan Neptune^68^. A gravimetrically calibrated^69^ triple-spike, ^229^Th-^233^U-^236^U, and the isotope dilution method were employed to correct for mass bias and to determine U-Th isotopic compositions and contents. Half-lives of U-Th nuclides used for U-Th age calculation are given in ref. 69. Uncertainties in isotopic data and dates, relative to 1950 C.E., are given at the two-sigma (2σ) level or two standard deviations of the mean (2σ_m_) unless otherwise noted. U-Th contents, isotopic compositions and ages are given in Supplementary Data 1. Age corrections for the initial ^230^Th are 0–2 years, smaller than dating errors of ± 2–30 years. A Monte-Carlo-derived age-depth model was constructed using StalAge^70^ techniques (Supplementary Fig. 5).

### Stable oxygen isotopes

A total of 732 (BA14-1) and 265 (BA18-4) powdered subsamples, 10–50 μg each, were micro-milled at 0.05–0.10 mm intervals along the growth axis (Supplementary Data 2 and Supplementary Figs. 4 and 6). Four to seven coeval subsamples of 13 layers were drilled for Hendy tests^20^. The oxygen isotopic composition was analyzed on Thermo-Finnigan MAT 253 mass spectrometers at the College of Geography Science, Nanjing Normal University, the Department of Geography Science, Fujian Normal University and the Department of Natural History Sciences, Faculty of Science, Hokkaido University. Subsamples for Hendy tests were analyzed on a Micromass IsoPrime mass spectrometer at the Department of Earth Sciences, National Taiwan Normal University. All δ^18^O values are reported in per mil (‰), relative to the Vienna PeeDee Belemnite (VPDB) and standardization was accomplished using NBS-19. Reproducibility of δ^18^O measurements was ± 0.08–0.12‰ at the 1-sigma level.

Average δ^18^O values of aragonitic BA14-1 are 1.2‰ higher than those of calcitic BA18-4 during the overlapping period from 702 to 752 yr BP (Supplementary Fig. 6). This offset falls within the range of 0.6–1.4‰ suggested by previous speleothem studies^71^. To combine the two series, the BA14-1 δ^18^O data was subtracted by 1.2‰ and attached to the BA18-4 δ^18^O series. The overlapping δ^18^O (702–752 yr BP) was averaged and resampled at 4-year intervals to match the average resolution of the overlapping δ^18^O. We also introduced an addition error of ± 0.2‰ derived from the one standard deviation of δ^18^O differences of BA14-1 and BA18-4 during the overlapping period. The maximum error in combination with the instrumental error (± 0.12‰) is ± 0.23‰ for the new time series. The composite Δ^18^O data (Supplementary Data 2; Supplementary Fig. 6) were then normalized to the mean δ^18^O value (–5.8‰) of the new series over the entire period.

### Carbonate Sr/Ca

Powdered subsamples, 10–50 μg each, were micro-milled at 0.1–0.5 mm intervals along the growth axis for Sr/Ca determination on BA14-1 and BA18-4, with external matrix-matched standards for every 4–5 samples on an inductively coupled plasma sector-field mass spectrometer, Finnigan Element II, at the HISPEC, National Taiwan University^72^. The 2-sigma reproducibility is ± 0.5%.

BA14-1 Sr/Ca, with a mean of 1.2 (± 0.2, 1σ) mmol/mol, vary from 0.45 to 1.9 mmol/mol. BA18-4 Sr/Ca range from 0.035 to 0.25 mmol/mol and the mean is 0.048 (± 0.017, 1σ) mmol/mol. The offset between the two Sr/Ca datasets is caused by different partition coefficients, 0.1–0.2 for calcite^73^ and 0.8–2.0 for aragonite^74^. To combine the two Sr/Ca series, BA14-1 and BA18-4 Sr/Ca data were first converted to z-standard records using the mean and standard deviation of each dataset. The converted z-standard records (Supplementary Data 3) were zigzagged in the overlapping interval, 728–875 yr BP, based on the assumption that the precipitation changes influenced Sr/Ca of aragonite (BA14-1) and calcite (BA18-4) in the same direction (Supplementary Text 3).

### Error propagation of correlation analysis

For correlations between data without age uncertainties, the upper/lower limits for the correlation coefficient were added, derived from the probable errors^75^ (P.E.) in correlations with P.E. = 0.6745 × (1−*r*^2^)/*N*^0.5^, where *r* is the correlation coefficient and *N* is the number of observation pairs.

For the correlation of two time series with age uncertainties, the method of Fohlmeister et al. (2012)^76^ was applied. This method embraces a Monte Carlo approach to simulate the best correlation between two series. The maximum correlation coefficient between two real series is presented in the text followed by the significance level. Specifically, more than two thousand artificial random time series that have the same characteristics as the original time series (e.g., variance, auto-correlation coefficients, data resolution and absolute ages with age uncertainties) were generated. By tuning these artificially generated time series, the distribution of the best correlations and the significant level can be obtained. For example, if 5% of tuned artificial series yield a correlation coefficient above 0.5, the best correlation of real series with a coefficient of > 0.5 is significant at the 95% significance level. The maximum correlation coefficient between two series is presented in the text followed by the significance level.

For Supplementary Table 2, the correlation analysis was conducted on centennial to multi-centennial scales. We first resampled each of the 20 proxy records, using 15-year time windows and averaged these values for consecutive 150-year periods, resulting in time series of X values (degrees of freedom = X–1), each representing a 150-year average. We then calculated correlation coefficients between each of these time series and the transformed Bàsura record.

### Community Earth System Model Version 2

A Community Earth System Model Version 2 (CESM2) preindustrial simulation^66^ was performed at approximately 2° × 2° resolution to represent natural climate variability. To verify that, the proposed processing holds on multidecadal to centennial time scales as in our records, a 5-yr running mean on this long preindustrial simulation was performed in a 300-year window. The sea-surface temperature data were correlated with the 700-mb height pressure variation in the eastern Atlantic (52˚30’N, 27˚30’W).

## Supplementary information

Supplementary Information

Description of Additional Supplementary Files

Supplementary Dataset 1

Supplementary Dataset 2

Supplementary Dataset 3

Supplementary Movie 1

## Data Availability

U-Th report, δ^18^O and Sr/Ca of BA14-1 and BA18-4 are available in the Source Data file.
