# Supplementary material for: Tracking westerly wind directions over Europe since the middle Holocene
Source: Nat Commun. 2022 Dec 21;13:7866. doi: 10.1038/s41467-022-34952-9 (PMC9772192; doi:10.1038/s41467-022-34952-9)
Supplement: Supplementary file 1 — Supplementary Information [file 41467_2022_34952_MOESM1_ESM.docx]

**Supplementary Information**

**Tracking westerly wind directions over Europe since the middle Holocene**

Hsun-Ming Hu, Valerie Trouet, Christoph Spötl, Hsien-Chen Tsai, Wei-Yi Chien, Wen-Hui Sung, Véronique Michel, Jin-Yi Yu, Patricia Valensi, Xiuyang Jiang, Fucai Duan, Yongjin Wang, Horng-Sheng Mii, Yu-Min Chou, Mahjoor Ahmad Lone, Chung-Che Wu, Elisabetta Starnini, Marta Zunino, Takaaki K. Watanabe, Tsuyoshi Watanabe, Huang-Hsiung Hsu, G.W.K. Moore, Giovanni Zanchetta, Carlos Pérez-Mejías, Shih-Yu Lee and

Chuan-Chou Shen

**This PDF file includes:**

Supplementary Text and References

Supplementary Tables 1 to 2

Supplementary Figures 1 to 15

Titles of Supplementary Data 1 to 3

Title of Supplementary Movie 1

**Supplementary data files:**

Supplementary Data 1: BA14-1 and BA18-4 U-Th dating report

Supplementary Data 2: Data of BA14-1 and BA18-4 δ^18^O values, ∆^18^O

Supplementary Data 3: Data of BA14-1 and BA18-4 Sr/Ca ratios

Supplementary Movie 1: Thirty-year window of running correlation between winter (December-February) precipitation and NAO index during 1836-2014 C.E based on NCAR/NCEP Reanalysis v3 (https://www.esrl.noaa.gov/). Maps were generated using KNMI climate explorer (https://climexp.knmi.nl/).

Supplementary Text

1. Geological setting

The Toirano cave system is located in the province of Savona, Italy (44˚08’ N, 8˚12’ E), consisting of Bàsura, Columbo, and Santa Lucia caves. Bàsura cave, about 200 m above sea level, is composed of two main passages connected with a 50-m artificial tunnel^1^ (Supplementary Fig. 3a). Two stalagmites, BA14-1 and BA18-4 (Supplementary Fig. 4), were collected for this study. The host rock is a Triassic limestone of Monte San Pietro (Briançon series) with dolomitic intervals. The vegetation comprises grasses with *Brachypodium ramosum* and *Tuberaria guttata* and forests consisting of *Pinus halepensis*, Lamiaceae shrubland, as well as multiple enclaves of olive groves. About 20% of the surface is covered by 0-20 cm-think soil (Supplementary Fig. 3d and e).

1. Interpretation of δ^18^O in stalagmites

The stalagmite oxygen isotope composition is controlled by both temperature and δ^18^O of the dripwater when the stalagmite deposited at near isotopic equilibrium conditions. The Hendy test^2^ (Methods) has been commonly used for isotopic equilibrium investigation; studies^3,4^ strongly suggested the necessity of replication test to verify the climatic significance of stalagmite δ^18^O. For the in-cave replication test, the overlapping period of our stalagmites is too short (707-752 yr BP) to conduct. The inter-cave replication test with stalagmite δ^18^O records from caves in the Alps, Italy, and Spain (Supplementary Fig. 9) indicates that the Bàsura stalagmite δ^18^O reflects the characteristics of meteoric precipitation.

Monitoring of the dripwater rate near the sites of BA14-1 and BA18-4 in Bàsura cave from April 2019 to February 2020 shows that the drip rate peak in December, which is a slightly delayed response to the precipitation peak in November. Dripwater rate records show a high correlation with monthly precipitation records, with *r* = 0.81 (*n* = 11, *p* < 0.1) at BA14-1 and *r* = 0.84 (*n* = 11, *p* < 0.1) at BA18-4, suggesting that the rate respond rather quickly to meteoric precipitation events. Evaporation and transpiration, however, cause the loss of over 50% of annual precipitation in Europe^5^. European stalagmites hence document recharge-weighted δ^18^O (i.e., winter precipitation δ^18^O, δ^18^O_p_)^6-9^. In the Mediterranean realm, up to 70% of the annual precipitation is lost by evapotranspiration^10^, suggesting that δ^18^O_p_ in rainy seasons predominately controls stalagmite δ^18^O.

The observational winter (December-February, DJF) European δ^18^O_p_ data, from the Global Network of Isotope in Precipitation (GNIP) under the International Atomic Energy Agency (IAEA)(https://www.iaea.org/services/networks/gnip), reveal a strong positive correlation with winter (DJF) temperature^9^, with 0.1–0.3‰ increase in δ^18^O_p_ per ˚C. Several studies, instead, suggested a small temperature effect on Mediterranean stalagmite δ^18^O^11,12^. This ostensible inconsistency is attributed to the fact that the temperature effect on δ^18^O_p_ (0.1–0.3‰ per ˚C) is approximately counterbalanced by the temperature effect of the water-carbonate oxygen isotope fractionation (~ –0.24‰ per ˚C)^13,14^. Instrumental data from the Genoa meteorological station shows a positive correlation (*r* = 0.54 ± 0.02, *n* = 326, *p* < 0.05; 1964–1995 C.E.) of 0.2 ± 0.02‰ per ˚C between Genoa δ^18^O_p_ and temperature. The local temperature effect here is likely counterbalanced by water-carbonate oxygen isotope fractionation and the stalagmite data reflect the “source effect” and “amount effect”^11,12,15,16^.

The “source effect” encompasses the influences from various moisture sources on δ^18^O_p_, which can be estimated using the deuterium excess (d-excess)^17,18^. High d-excess values usually reflect low humidity in the semi-close oceanic source region due to substantial kinetic isotope fractionation during fast evaporation. Moisture sourced from the Mediterranean Sea hence features high d-excess values of ~22‰ (ref. 18) compared to Atlantic-sourced moisture with low values of ~10‰ (ref. 19). An average annual precipitation d-excess value of 11.1 ± 2.5‰ (*n* = 90; 1961–2012 C.E.) from 266 meteorological stations in northern Italy^20^ and of 10.0 ± 2.1‰ (1962–1995 C.E.; *n* = 27) in Genoa station suggests predominantly Atlantic-sourced precipitation rather than Mediterranean-sourced in the study region. The DJF d-excess values of Genoa rainwater indeed show no significant correlation with DJF precipitation amount, NAO index^21^, and the CPC East Atlantic (EA) index (1962–1995 C.E.), suggesting that the effect of changing moisture sources on rainwater composition is not clear. Wind direction analysis (1985–2015 C.E.; data from Meteoblue, University of Basel, Switzerland) expresses ~50% land breezes and ~20% sea breezes at Toirano, suggesting less contribution of Mediterranean-sourced rainwater. These evidences show that the moisture source in Toirano region is mainly from Atlantic, suggesting that the alteration of moisture source regions could not dominate the δ^18^O_p_. The change in δ^18^O of surface water in Atlantic/Mediterranean could also contribute to the source effect. However, the low-resolved existing records hamper further investigation while this might be relatively small on decadal to centennial scales^22^.

The “amount effect” infers a negative relationship between rainfall amount and δ^18^O_p_ owing to the extent of vapor-water δ^18^O fractionation in air mass^17^. Instrumental data show a correlation coefficient of –0.29 between monthly Genoa δ^18^O_p_ data and rainfall amount (*n* = 324, *p* < 0.1; 1964–1995 C.E.) with a gradient of –0.70 ± 0.13‰ per 100 mm. Stronger westerlies usually lead to more precipitation and more Atlantic-sourced rainwater, resulting in more negative δ^18^O_p_. An exception are Genova cyclones, the most frequent winter cyclone type originating in the Gulf of Genova^23^, which leads to intense rainfall of Mediterranean-sourced moisture in the study area. However, the cyclones could result in extreme negative δ^18^O_p_ of –11 to –23‰ (ref. 24) due to a strong amount effect. Since the δ^18^O_p_ difference between Atlantic-sourced δ^18^O_p_ (–8.5‰) and Mediterranean-soured δ^18^O_p_ (–4.6‰) is only ~4‰ (ref. 24), the strong Mediterranean-sourced precipitation events caused by Genoa cyclones hardly result in an increase of δ^18^O_p_. In fact, the intensity and frequency of Genoa cyclones are strongly related to the strength of the Azores High^25^, which controls the westerlies in Europe. Accordingly, the source effect on local δ^18^O_p_ is hence relatively small and Mediterranean stalagmite δ^18^O can be in general considered to be governed by rainfall amount, consistent with the previous studies^11,12,26-30^.

Baldini et al.^9^ showed a correlation of 0.41 between the NAO index^21^ and Genoa δ^18^O_p_ and –0.74 between the NAO index^21^ and Genoa precipitation amount from December to March during 1962–1995 C.E. This suggests that the Genoa δ^18^O_p_ could be modulating in a negative way by southerly shifted westerlies and associated increasing precipitation (or/and decreasing temperature) under the negative NAO index. Surprisingly, stalagmite BA18-4 δ^18^O reveals a correlation of –0.46 (*n* = 85, 95%) with the winter (DJF) NAO index^21^ from 1825-1979 C.E., suggesting the studied region was instead located in a positive-NAO correlated region at this time window. We argue that the variable correlations between Genoa δ^18^O_p_ (or stalagmite δ^18^O) and the NAO index^21^ (r = 0.41 from 1962–1995 C.E.; *r* = –0.45 from 1825–1979 C.E) can be attributed to the non-stationary NAO behavior (Supplementary Movie 1), i.e., the migration of the Azores High and Icelandic Low that changes the NAO-affected region in mainland Europe^31^. An unclear correlation between Toirano precipitation and the NAO index in the early 20^th^ century (Fig. 2c, blue line) also suggests that the Toirano region was temporally located in the positive NAO-correlated region. Taken together, these observations suggest that δ^18^O_p_ and eventually stalagmite δ^18^O, via modulation by the “amount effect” and minor “source effect,” reflect the westerly variability and the large-scale atmospheric circulation, with negative/positive δ^18^O values corresponding to strong/weak westerlies and high/low rainfall amount, partly supported by the Sr/Ca data (Supplementary Text 3; Supplementary Data 3).

1. Interpretation of stalagmite Sr/Ca

Stalagmite Sr/Ca could vary under different regional hydroclimatic and cave conditions^32^. The processes include incongruent weathering of carbonate source, selective leaching of Sr from minerals, dissolution of dolomite, stalagmite crystallography, in addition to hydroclimate controls^32-37^. In general, stalagmite Sr/Ca can be considered as an indicator of cave hydrology variations via the mechanism of prior calcite precipitation (PCP)^32-35^. Dry/wet conditions are expected to enhance/reduce CO_2_ degassing and result in a long/short residence time of the infiltrated water in the epikarst and promote/depress PCP, leading to high/low Sr/Ca in dripwater and stalagmite. The reported Sr/Ca water-carbonate distribution coefficient (D_Sr_), (Sr/Ca)_carbonate_ /(Sr/Ca)_water_ , ranges from 0.1–0.2 for calcite^33^ and 0.8–2.0 for aragonite^34^. This could consequently lead to both negative (D_Sr_ < 1) and positive (D_Sr_ > 1) aragonite Sr/Ca shifts in response to hydroclimate. Hence, our aragonitic BA14-1 Sr/Ca data cannot be regarded as an indicator of effective precipitation. The pattern of BA14-1 Sr/Ca, however, shows some similarity with δ^18^O (Supplementary Fig. 8), with a correlation coefficient of 0.4 ± 0.02 (*n* = 368, *p* < 0.1). Since precipitation source changes cannot alter Sr/Ca, the covariation between Sr/Ca and δ^18^O suggests that precipitation amount could have simultaneously driven Sr/Ca and δ^18^O in Bàsura stalagmites.

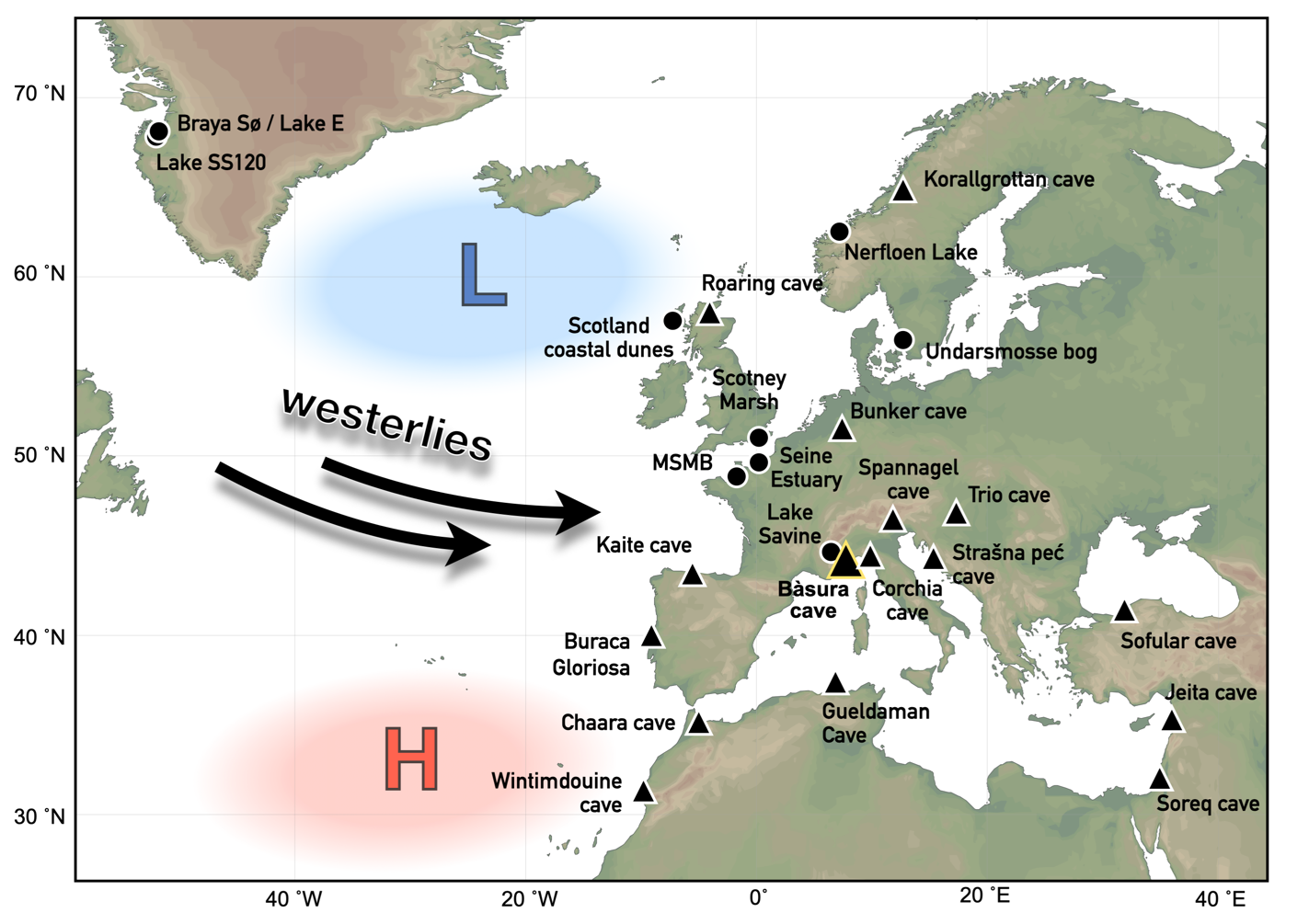


**Supplementary Figure 1.** **Map of the study area.** Triangles (caves) and circles (lakes) are the sites mentioned in main text. Pink and cyan zones denote the conceptual position of high-pressure (Azores High) and low-pressure (Icelandic Low) centers during boreal winter (December-February) based on NCAR/NCEP Reanalysis v3 (https://www.esrl.noaa.gov/) from 1950-2019 C.E. Arrows show the prevailing westerly winds. This map was generated using Ocean Data View. The quoted sites are listed in Supplementary Table 1.


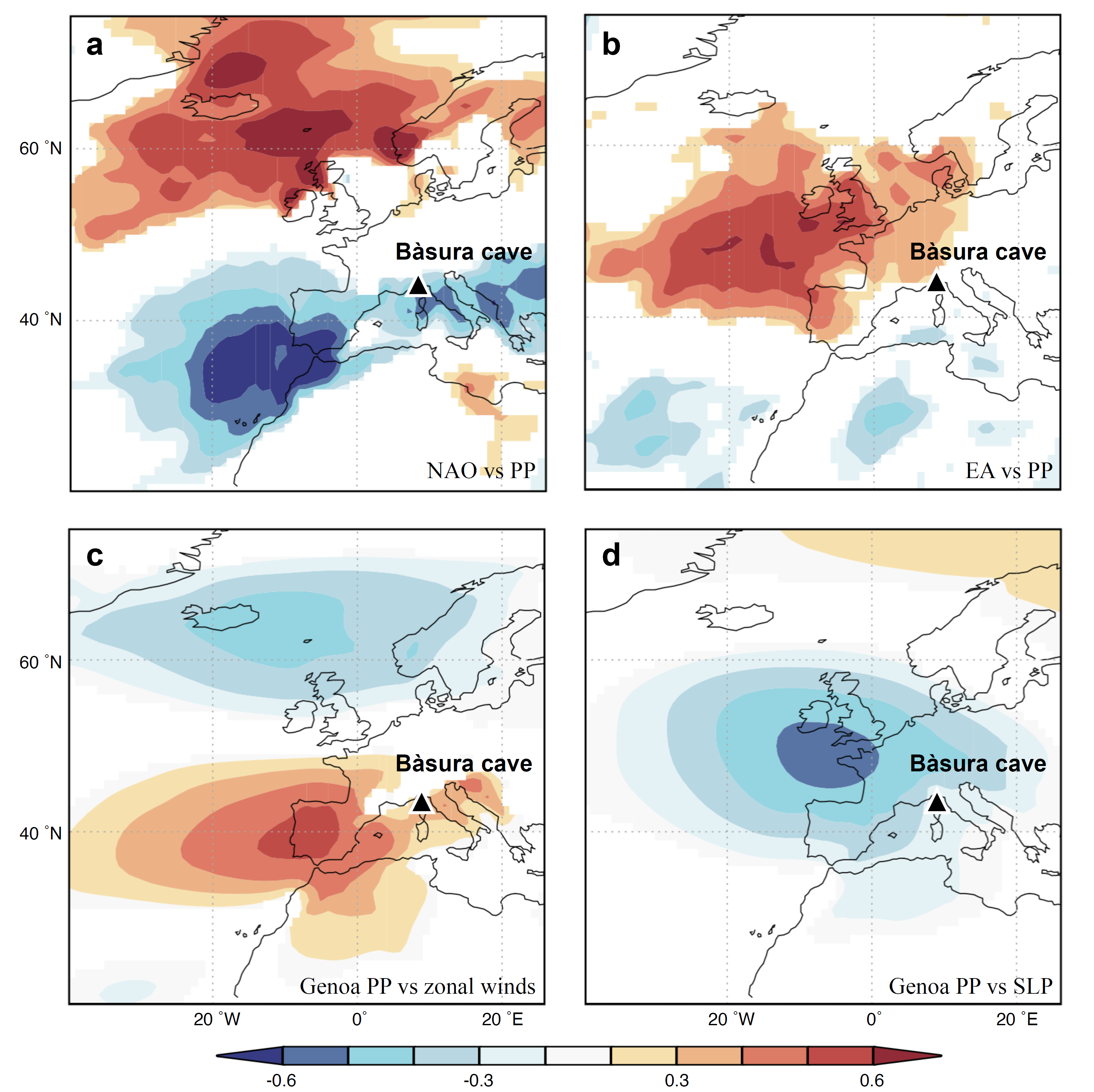


**Supplementary Figure 2.** **Regional climate dynamics. a** Correlation of winter (December-February) precipitation and NAO index^21^ during 1950–2019 C.E. A positive/negative NAO index indicates large/small pressure contrast between Icelandic Low and Azores High. **b** as **a**, but with East Atlantic (EA) index (data from Climate Predict Center, https://www.cpc.ncep.noaa.gov/data/teledoc/ea.shtml). The positive/negative EA index corresponds to low/high pressure anomaly near 52.5˚N, 27.5˚W. **c** Correlation of September-February (rainy season) precipitation at the Genoa station with 850 mb zonal wind speed during 1836–2008 C.E. **d** as **c**, but with sea-level pressure (SLP). The correlation coefficient is shown by the color bar. All correlations are significant at 90% confidence level. (Climate data from NCAR/NCEP Reanalysis v3, https://www.esrl.noaa.gov/). Maps are generated using KNMI climate explorer (https://climexp.knmi.nl/).

Supplementary Figure 3. Location and images of Bàsura cave. a Locations of Bàsura cave and the Genoa meteorological station. This map was generated using Ocean Data View. b Simplified plan view of the cave with the stalagmite sampling sites (stars). Dashed lines represent a 50-m artificial tunnel, connecting two main passages. c A chamber in Bàsura cave. d and e are photographs out of cave.


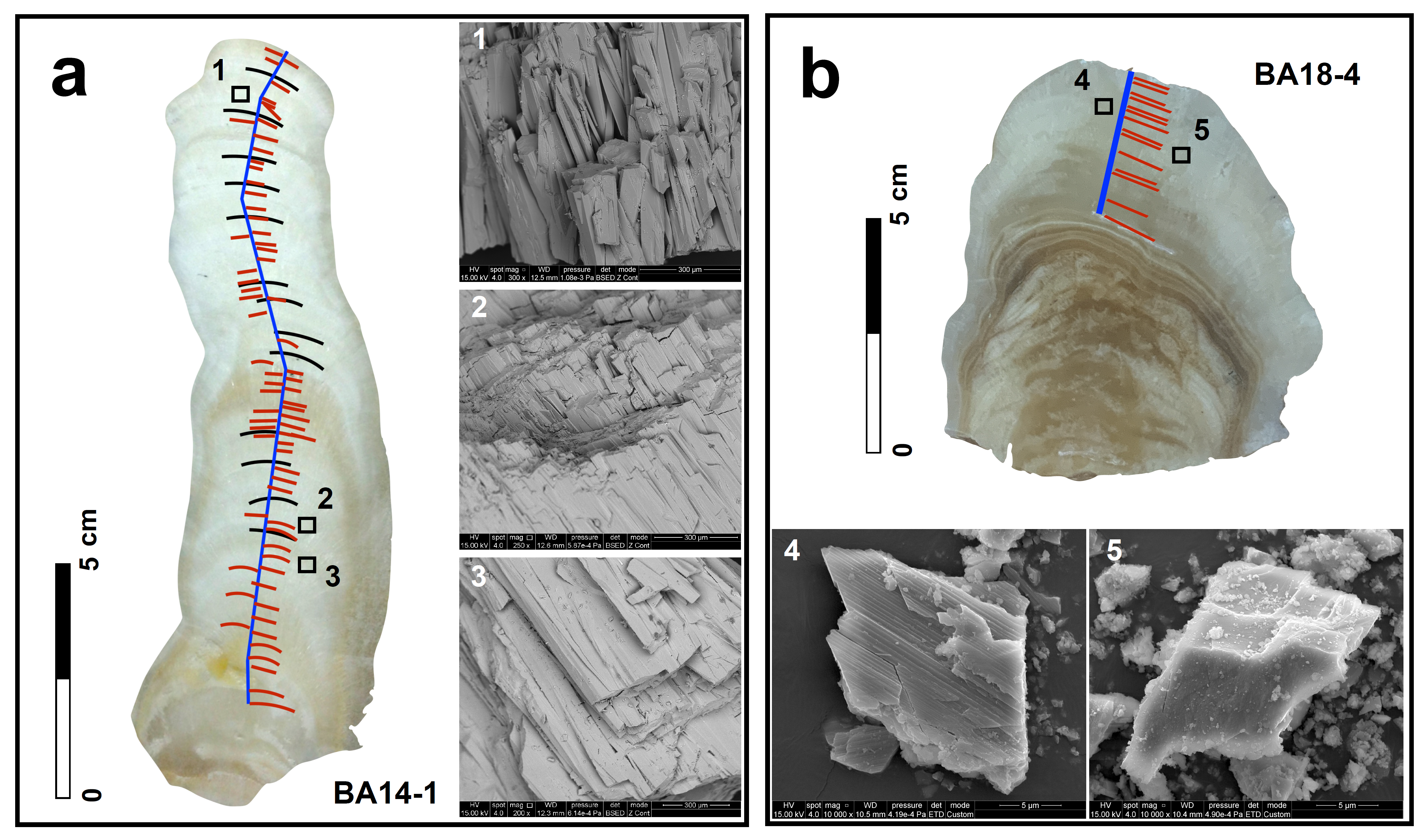


Supplementary Figure 4. Stalagmites BA14-1 and BA18-4. a Polished halved stalagmite BA14-1 and the scanning electron microscopic images, showing needle-shaped aragonite crystals at three arbitrarily selected layers (1, 2 and 3). b as a, but for BA18-4 with scanning electron microscopic images of calcite crystals and their rhombohedral cleavages at two arbitrarily selected layers (4 and 5). Black and red lines respectively indicate layers for Hendy test^2^ and U-Th dating. Subsamples for measurements of oxygen isotope and Sr/Ca ratios with different resolution were drilled from the central deposition axis (blue line).

Supplementary Figure 5. Stalagmite chronology. Age models for stalagmites a BA14-1 and b BA18-4 were built with ^230^Th dates by StalAge methods^38^. Black solid and red dashed lines mark the chronology and 2-sigma envelopes, respectively.

Supplementary Figure 6. Bàsura stalagmite δ^18^O series. a BA18-4 δ^18^O series. b BA14-1 δ^18^O series. c The composite ∆^18^O series.


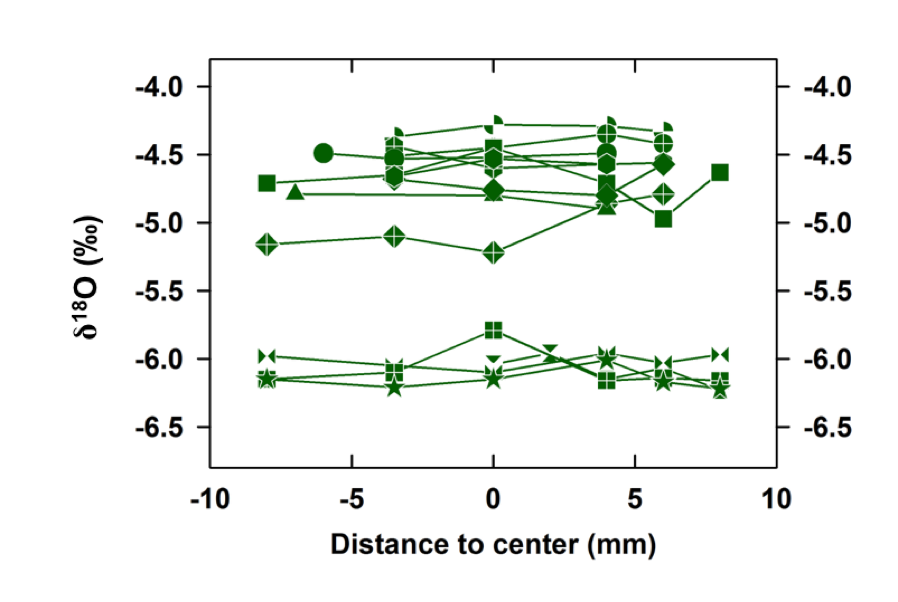


Supplementary Figure 7. Hendy test. Hendy test^2^ on 13 individual layers of stalagmite BA14-1 (Supplementary Fig. 4a). The small two-sigma variability of ± 0.08–0.21‰ in δ^18^O for coeval subsamples of the same growth layers suggests negligible kinetic isotope fractionation.


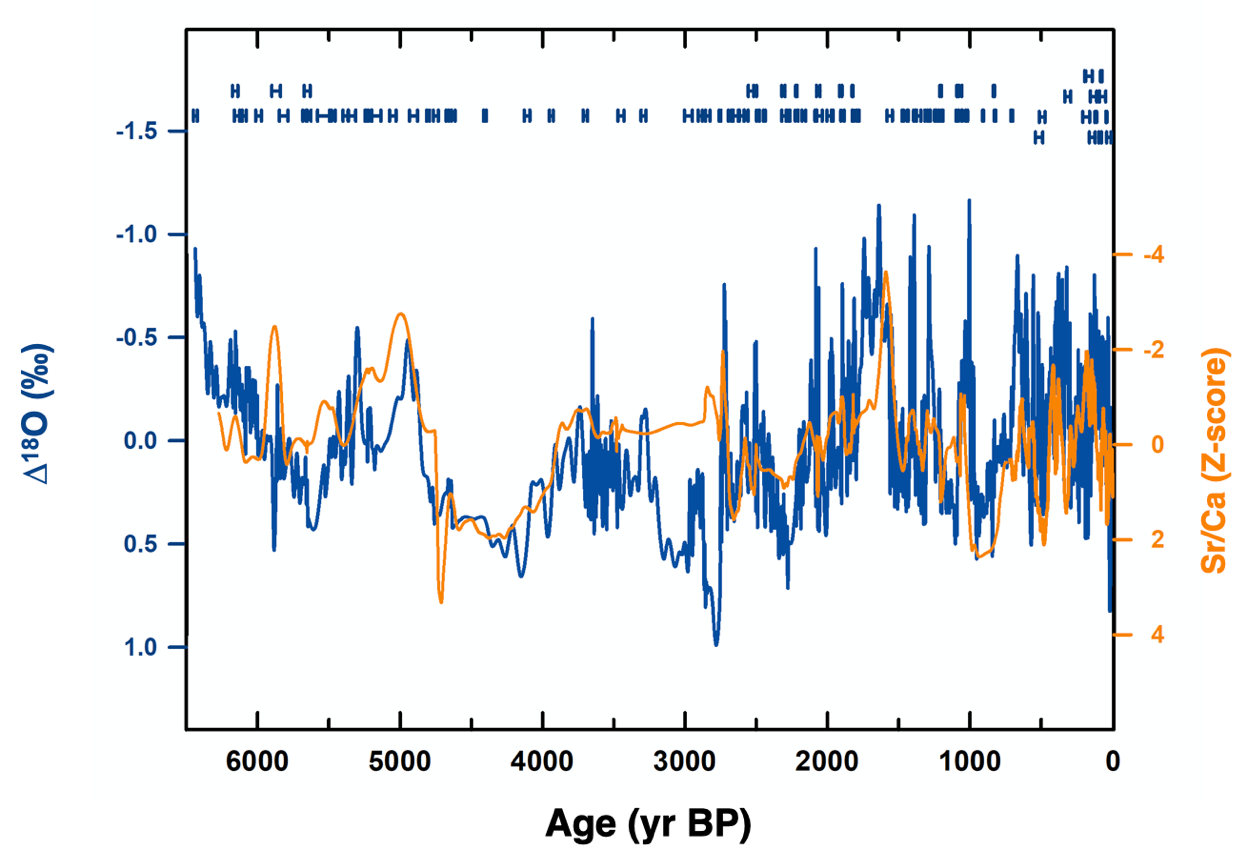


Supplementary Figure 8. Bàsura Δ^18^O and Sr/Ca records. Comparisons of Δ^18^O (dark blue) and Z-score of Sr/Ca (orange). Blue bars indicate ^230^Th ages with 2-sigma errors.

Supplementary Figure 9. Comparison of Bàsura ∆^18^O record with other proxy records from the western Mediterranean region. a Flood record from Lake Savine, northern Italy^39^. b Stalagmite trace element-inferred rainfall index record from Corchia cave, northern Italy^40^. c Stalagmite uranium concentrations from Corchia cave, northern Italy^41^. d Stalagmite δ^18^O record from Kaite cave, Spain^15^. e Stalagmite δ^18^O record from Buraca Gloriosa, Portugal^26^. The inconsistency of Bàsura and Buraca Gloriosa records during 1.4-1.8 kyr BP could be attributed to the age uncertainties or resampling biases of this composite Gloriosa records. f Stalagmite δ^18^O record from Gueldaman GLD1 cave, Algeria^27^. All dark blue lines express Bàsura ∆^18^O record. The light-blue shadow backgrounded Bàsura ∆^18^O shows the ranges of 2-sigma age uncertainties. Upward trend of all records indicates wet/warm climate. Cyan/pink shaded areas denote the intervals of 5.4–3.5 and 2.2–1.2 kyr BP.

**Supplementary Figure 10.** **Comparison of Bàsura Δ^18^O** **with other stalagmite proxy records from the eastern Mediterranean and northern Africa. a** δ^13^C record from Sofular cave, Turkey^42^. **b** δ^18^O record from Jeita cave, Lebanon^28^. **c** δ^18^O record from Soreq cave, Israel^29^. **d** Speleothem δ^18^O-based rainfall index record from Charra cave, northern Africa^43^. **e** δ^18^O record from Wintimdouine cave, Morocco^44^. All dark blue lines express Bàsura ∆^18^O record. The light-blue shadow backgrounded Bàsura ∆^18^O shows the ranges of 2-sigma age uncertainties. Upward trend of all records indicates wet/warm climate. Cyan/pink shaded areas denote the intervals of 5.4–3.5 and 2.2–1.2 kyr BP.

**Supplementary Figure 11.** **Comparison of Bàsura ∆^18^O with other stalagmite proxy records from central and northern Europe. a** δ^18^O record from Korallgrottan cave, Sweden^45^. **b** Growth rate-based precipitation record from Roaring cave, Scottland^46^. **c** δ^18^O record from Trio cave, Hungary^47^. **d** δ^18^O record from Strašna peć cave, Croatia^48^. All dark blue lines express Bàsura ∆^18^O record. The light-blue shadow backgrounded Bàsura ∆^18^O shows the ranges of 2-sigma age uncertainties. Upward trend of all records indicates wet/warm climate. Cyan/pink shaded areas denote the intervals of 5.4–3.5 and 2.2–1.2 kyr BP.

**Supplementary Figure 12.** **Bàsura ∆^18^O record with timing of Mediterranean cultural events.** Dark blue horizontal bars denote ^230^Th dates with 2-sigma errors. Black numbers highlight prominent dry periods at 5.2 and 4.8–4.1 kyr BP. Light brown arrows show the timing of cultural demise in Mesopotamia/Egypt.

**Supplementary Figure 13.** **Reanalysis data. a** Leading empirical orthogonal function (EOF) 1 of Sep–Feb precipitation, representing an East Atlantic (EA) teleconnection. **b** EOF2 of Sep–Feb precipitation, showing a North Atlantic Oscillation (NAO) teleconnection. Data source: NCAR/NCEP Reanalysis v3 (https://www.esrl.noaa.gov/), 1836–2008 C.E. Maps were generated using KNMI climate explorer (https://climexp.knmi.nl/).

**
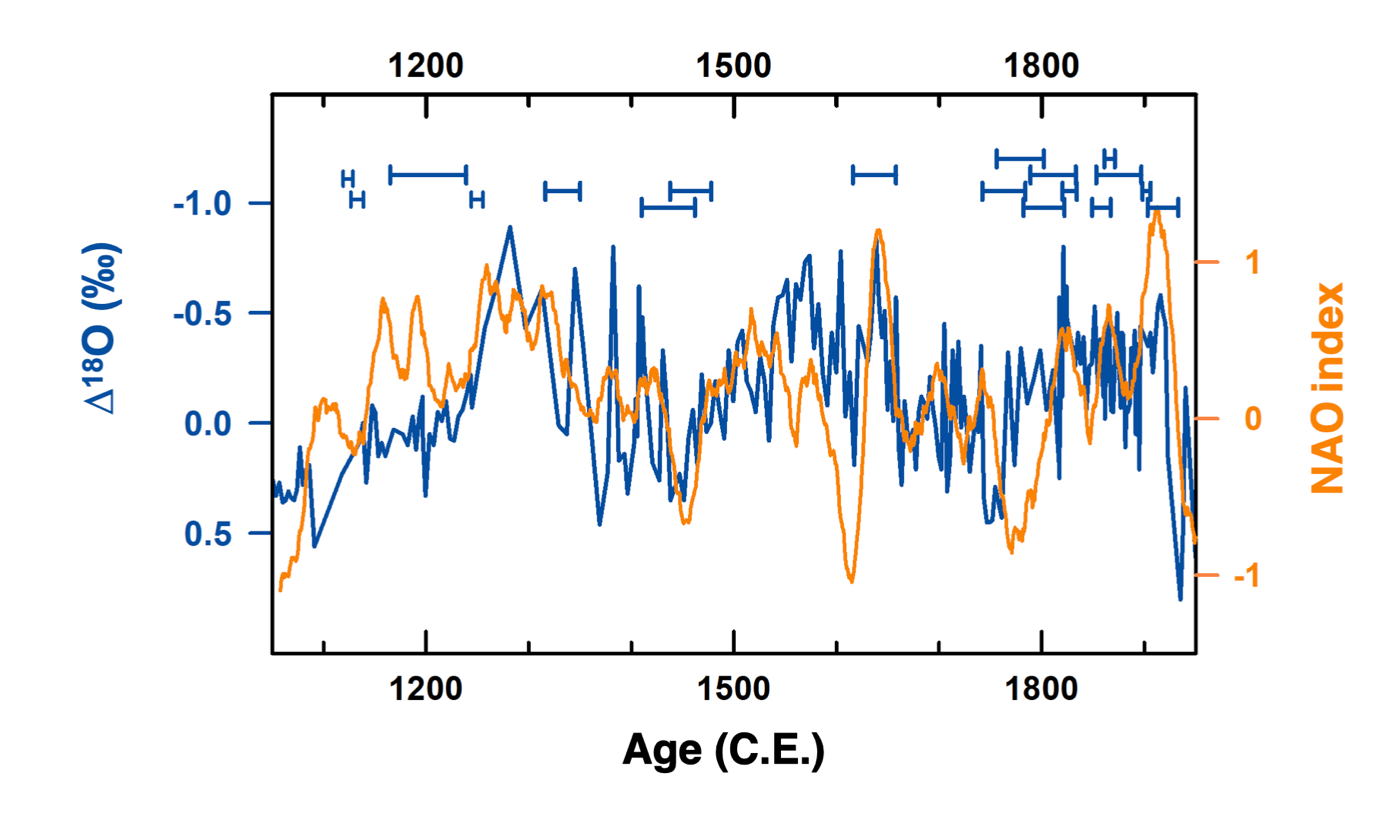
**

**Supplementary Figure 14. Bàsura ∆^18^O and North Atlantic Oscillation (NAO).** Comparison of Bàsura ∆^18^O record (dark blue) and 19-year moving average NAO index^49^ (orange) from 1049–1969 C.E. Noted the correlation coefficient between ∆^18^O-inferred paleo-rainfall and NAO that is reported in the main text is inverted due to the anti-phased relationship between precipitation and ∆^18^O record. Horizontal bars denote ^230^Th dates with 2-sigma errors for Bàsura ∆^18^O record.

**Supplementary Figure 15. Shift of precipitation domains over Europe.** Three-hundred-year running correlation analysis between the Bàsura record and **a** North Atlantic Oscillation (NAO) index^50^, **b** principal component analysis (PCA) result based on glacier activity reconstructions from alpine lake sediments in Norway^51^, **c** stalagmite δ^18^O records from Bunker cave, Germany^52^, and **d** stalagmite δ^18^O from Spannagel cave, Austria^53^.

**Supplementary References**

1. Columbu, A. *et al.* Hypogenic speleogenesis, late stage epigenic overprinting and condensation-corrosion in a complex cave system in relation to landscape evolution (Toirano, Liguria, Italy). *Geomorphology* **376**, 107561 (2021).
2. Hendy, C. The isotopic geochemistry of speleothems—I. The calculation of the effects of different modes of formation on the isotopic composition of speleothems and their applicability as palaeoclimatic indicators. *Geochim. Cosmochim. Acta* **35**, 801–824 (1971).
3. Mühlinghaus, C., Scholz, D. & Mangini, A. Modelling fractionation of stable isotopes in stalagmites. *Geochim. Cosmochim. Acta* **73**, 7275–7289 (2009).
4. Dorale, J. A. & Liu, Z. Limitations of Hendy test criteria in judging the paleoclimatic suitability of speleothems and the need for replication. *J. Cave Karst Stud.* **71**, 73–80 (2009).
5. Wackerbarth, a. *et al.* Simulated oxygen isotopes in cave drip water and speleothem calcite in European caves. *Clim. Past* **8**, 1781–1799 (2012).
6. Baker, A. *et al.* Global analysis reveals climatic controls on the oxygen isotope composition of cave drip water. *Nat. Commun.* **10**, 1–7 (2019).
7. Fohlmeister, J. *et al.* Bunker Cave stalagmites: An archive for central European Holocene climate variability. *Clim. Past* **8**, 1751–1764 (2012).
8. Wassenburg, J. A. *et al.* Reorganization of the North Atlantic Oscillation during early Holocene deglaciation. *Nat. Geosci.* **9**, 6–11 (2016).
9. Baldini, L. M., McDermott, F., Foley, A. M. & Baldani, J. U. L. Spatial variability in the European winter precipitation δ^18^O-NAO relationship: Implications for reconstructing NAO-mode climate variability in the Holocene. *Geophys. Res. Lett.* **35**, L04709 (2008).
10. Moussa, R., Chahinian, N. & Bocquillon, C. Distributed hydrological modelling of a Mediterranean mountainous catchment - Model construction and multi-site validation. *J. Hydrol.* **337**, 35–51 (2007).
11. Regattieri, E. *et al.* Holocene Critical Zone dynamics in an Alpine catchment inferred from a speleothem multiproxy record: disentangling climate and human influences. *Sci. Rep.* **9**, 1–9 (2019).
12. Moreno, A. *et al.* New speleothem data from Molinos and Ejulve caves reveal Holocene hydrological variability in northeast Iberia. *Quat. Res.* **88**, 223–233 (2017).
13. O’Neil, J. R., Clayton, R. N. & Mayeda, T. K. Oxygen isotope fractionation in divalent metal carbonates. *J. Chem. Phys.* **51**, 5547–5558 (1969).
14. Johnston, V. E., Borsato, A., Spötl, C., Frisia, S. & Miorandi, R. Stable isotopes in caves over altitudinal gradients: Fractionation behaviour and inferences for speleothem sensitivity to climate change. *Clim. Past* **9**, 99–118 (2013).
15. Domínguez-Villar, D., Wang, X., Krklec, K., Cheng, H. & Edwards, R. L. The control of the tropical North Atlantic on Holocene millennial climate oscillations. *Geology* **45**, 303–306 (2017).
16. Baldini, L. M. *et al.* North Iberian temperature and rainfall seasonality over the Younger Dryas and Holocene. *Quat. Sci. Rev.* **226**, 105998 (2019).
17. Dansgaard, W. Stable isotopes in precipitation. *Tellus* **16**, 436–468 (1964).
18. Gat, J. R. & Carmi, I. Evolution of the isotopic composition of atmospheric waters in the Mediterranean Sea area. *J. Geophys. Res.* **75**, 3039–3048 (1970).
19. Craig, H. Isotopic variations in meteoric waters. *Science* **133**, 1702–1703 (1961).
20. Giustini, F., Brilli, M. & Patera, A. Mapping oxygen stable isotopes of precipitation in Italy. *J. Hydrol. Reg. Stud.* **8**, 162–181 (2016).
21. Jones, P. D., Jonsson, T. & Wheeler, D. Extension to the North Atlantic Oscillation using early instrumental pressure observations from Gibraltar and south-west Iceland. *Int. J. Climatol.* **17**, 1433–1450 (1997).
22. Essallami, L., Sicre, M.-A., Kallel, N., Labeyrie, L. & Siani, G. Hydrological changes in the Mediterranean Sea over the last 30,000 years. *Geochem. Geophys. Geosy.* **8**, Q07002 (2007).
23. Campins, J., Genovés, A., Picornell, M. A. & Jansà, A. Climatology of Mediterranean cyclones using the ERA-40 dataset. *Int. J. Climatol.* **31**, 1596–1614 (2011).
24. Celle-Jeanton, H., Travi, Y. & Blavoux, B. Isotopic typology of the precipitation in the Western Mediterranean Region at three different time scales. *Geophys. Res. Lett.* **28**, 1215–1218 (2001).
25. Anagnostopoulou, C., Tolika, K., Flocas, H. & Maheras, P. Cyclones in the Mediterranean region: Present and future climate scenarios derived from a general circulation model (HadAM3P). *Adv. Geosci.* **7**, 9–14 (2006).
26. Thatcher, D. L. *et al.* Hydroclimate variability from western Iberia (Portugal) during the Holocene: Insights from a composite stalagmite isotope record. *The Holocene* **30**, 966–981 (2020).
27. Ruan, J. *et al.* Evidence of a prolonged drought ca. 4200 yr BP correlated with prehistoric settlement abandonment from the Gueldaman GLD1 Cave, Northern Algeria. *Clim. Past* **12**, 1–4 (2016).
28. Cheng, H. *et al.* The climate variability in northern Levant over the past 20,000 years. *Geophys. Res. Lett.* **42**, 8641–8650 (2015).
29. Bar-Matthews, M. & Ayalon, a. Mid-Holocene climate variations revealed by high-resolution speleothem records from Soreq Cave, Israel and their correlation with cultural changes. *The Holocene* **21**, 163–171 (2011).
30. Bard, E., Antonioli, F. & Silenzi, S. Sea-level during the penultimate interglacial period based on a submerged stalagmite from Argentarola Cave (Italy). *Earth Planet. Sci. Lett.* **196**, 135–146 (2002).
31. Comas-Bru, L. & Mcdermott, F. Impacts of the EA and SCA patterns on the European twentieth century NAO-winter climate relationship. *Q. J. R. Meteorol. Soc.* **140**, 354–363 (2014).
32. Fairchild, I. J. & McMillan, E. A. Speleothems as indicators of wet and dry periods. *Int. J. Speleol.* **36**, 69–74 (2007).
33. Day, C. C. & Henderson, G. M. Controls on trace-element partitioning in cave-analogue calcite. *Geochim. Cosmochim. Acta* **120**, 612–627 (2013).
34. Wassenburg, J. A. *et al.* Determination of aragonite trace element distribution coefficients from speleothem calcite–aragonite transitions. *Geochim. Cosmochim. Acta* **190**, 347–367 (2016).
35. Ronay, E. R., Breitenbach, S. F. M. & Oster, J. L. Sensitivity of speleothem records in the Indian Summer Monsoon region to dry season infiltration. *Sci. Rep.* **9**, 1–10 (2019).
36. Tremaine, D. M. & Froelich, P. N. Speleothem trace element signatures: A hydrologic geochemical study of modern cave dripwaters and farmed calcite. *Geochim. Cosmochim. Acta* **121**, 522–545 (2013).
37. Stoll, H. M., Müller, W. & Prieto, M. I-STAL, a model for interpretation of Mg/Ca, Sr/Ca and Ba/Ca variations in speleothems and its forward and inverse application on seasonal to millennial scales. *Geochem. Geophys. Geosy.* **13**, 1–27 (2012).
38. Scholz, D. & Hoffmann, D. L. StalAge – An algorithm designed for construction of speleothem age models. *Quat. Geochronol.* **6**, 369–382 (2011).
39. Sabatier, P. *et al.* 6-kyr record of flood frequency and intensity in the western Mediterranean Alps – Interplay of solar and temperature forcing. *Quat. Sci. Rev.* **170**, 121–135 (2017).
40. Regattieri, E. *et al.* Lateglacial to Holocene trace element record (Ba, Mg, Sr) from Corchia Cave (Apuan Alps, central Italy): Paleoenvironmental implications. *J. Quat. Sci.* **29**, 381–392 (2014).
41. Isola, I. *et al.* The 4.2 ka BP event in the Central Mediterranean: New data from Corchia speleothems (Apuan Alps, central Italy). *Clim. Past Discuss.* 1–24 (2018). doi:10.5194/cp-2018-127.
42. Fleitmann, D. *et al.* Timing and climatic impact of Greenland interstadials recorded in stalagmites from northern Turkey. *Geophys. Res. Lett.* **36**, L19707 (2009).
43. Ait Brahim, Y. *et al.* North Atlantic ice-rafting, ocean and atmospheric circulation during the Holocene: Insights from western Mediterranean speleothems. *Geophys. Res. Lett.* **46**, 7614–7623 (2019).
44. Sha, L. *et al.* How far north did the African monsoon fringe expand during the African humid period? Insights from southwest Moroccan speleothems. *Geophys. Res. Lett.* **46**, 14093–14102 (2019).
45. Sundqvist, H. S., Holmgren, K., Moberg, A., Spötl, C. & Mangini, A. Stable isotopes in a stalagmite from NW Sweden document environmental changes over the past 4000 years. *Boreas* **39**, 77–86 (2010).
46. Baker, A., C. Hellstrom, J., Kelly, B. F. J., Mariethoz, G. & Trouet, V. A composite annual-resolution stalagmite record of North Atlantic climate over the last three millennia. *Sci. Rep.* **5**, 10307 (2015).
47. Demény, A. *et al.* Middle Bronze Age humidity and temperature variations, and societal changes in East-Central Europe. *Quat. Int.* **504**, 80–95 (2019).
48. Lončar, N., Bar-Matthews, M., Ayalon, A., Faivre, S. & Surić, M. Holocene climatic conditions in the Eastern Adriatic recorded in stalagmites from Strašna peć Cave (Croatia). *Quat. Int.* **508**, 98-106 (2018).
49. Ortega, P. *et al.* A model-tested North Atlantic Oscillation reconstruction for the past millennium. *Nature* **523**, 71–74 (2015).
50. Olsen, J., Anderson, N. J. & Knudsen, M. F. Variability of the North Atlantic Oscillation over the past 5,200 years. *Nat. Geosci.* **5**, 808–812 (2012).
51. Vasskog, K., Paasche, Ø., Nesje, A., Boyle, J. F. & Birks, H. J. B. A new approach for reconstructing glacier variability based on lake sediments recording input from more than one glacier. *Quat. Res.* **77**, 192–204 (2012).
52. Fohlmeister, J. *et al.* Bunker Cave stalagmites: An archive for central European Holocene climate variability. *Clim. Past* **8**, 1751–1764 (2012).
53. Fohlmeister, J., Vollweiler, N., Spötl, C. & Mangini, A. COMNISPA II: Update of a mid-European isotope climate record, 11 ka to present. *Holocene* **23**, 749–754 (2013).
