# Supplementary material for: Tracking westerly wind directions over Europe since the middle Holocene
Source: Nat Commun. 2022 Dec 21;13:7866. doi: 10.1038/s41467-022-34952-9 (PMC9772192; doi:10.1038/s41467-022-34952-9)
Supplement: Supplementary file 2 — Description of Additional Supplementary Files [file 41467_2022_34952_MOESM2_ESM.pdf]

Description of Additional Supplementary Files:

Supplementary Data 1: BA14-1 and BA18-4 U-Th dating report

Supplementary Data 2: Data of BA14-1 and BA18-4 δ18O values, Δ18O

Supplementary Data 3: Data of BA14-1 and BA18-4 Sr/Ca ratios

Supplementary Movie 1: Thirty-year window of running correlation between winter (December-February) precipitation and NAO index during 1836-2014 C.E based on NCAR/NCE
P
Reanalysis v3 (https://www.esrl.noaa.gov/)
Maps were generated using KNMI climate explorer (https://climexp.knmi.nl/).
